# Neuropsychology of Environmental Navigation in Humans: Review and Meta-Analysis of fMRI Studies in Healthy Participants

**DOI:** 10.1007/s11065-014-9247-8

**Published:** 2014-02-01

**Authors:** Maddalena Boccia, Federico Nemmi, Cecilia Guariglia

**Affiliations:** 1 Dipartimento di Psicologia, Università La Sapienza, Via dei Marsi, 78, 00185 Rome, Italy; 2 Unità di Neuropsicologia, Fondazione Santa Lucia, Rome, Italy; 3 INSERM, U825, Université Paul-Sabatier, Toulouse, France

**Keywords:** fMRI, Human navigation, Meta-analysis, Frame of reference, Experimental paradigm

## Abstract

In the past 20 years, many studies in the cognitive neurosciences have analyzed human ability to navigate in recently learned and familiar environments by investigating the cognitive processes involved in successful navigation. In this study, we reviewed the main experimental paradigms and made a cognitive-oriented meta-analysis of fMRI studies of human navigation to underline the importance of the experimental designs and cognitive tasks used to assess navigational skills. We performed a general activation likelihood estimation (ALE) meta-analysis of 66 fMRI experiments to identify the neural substrates underpinning general aspects of human navigation. Four individual ALE analyses were performed to identify the neural substrates of different experimental paradigms (i.e., familiar vs. recently learned environments) and different navigational strategies (allocentric vs. egocentric). Results of the general ALE analysis highlighted a wide network of areas with clusters in the occipital, parietal, frontal and temporal lobes, especially in the parahippocampal cortex. Familiar environments seem to be processed by an extended temporal-frontal network, whereas recently learned environments require activation in the parahippocampal cortex and the parietal and occipital lobes. Allocentric strategy is subtended by the same areas as egocentric strategy, but the latter elicits greater activation in the right precuneus, middle occipital lobe and angular gyrus. Our results suggest that different neural correlates are involved in recalling a well-learned or recently acquired environment and that different networks of areas subtend egocentric and allocentric strategies.

**Electronic supplementary material:**

The online version of this article (doi:10.1007/s11065-014-9247-8) contains supplementary material, which is available to authorized users.

## Introduction

In the past 20 years, an increasing number of studies in the cognitive neuroscience literature have analyzed human ability to navigate and orient in recently learned and familiar environments by investigating the cognitive processes involved in successful navigation. The latter include the ability to retain the spatial layout of an environment, find a shortcut between two locations or create an interconnected network among different paths.

The data reported in these studies are often contrasting, perhaps because of differences in methods and in the specific cognitive processes investigated in the experimental paradigms. In this light, a methodological review with a meta-analytic approach could be useful to bridge the gap in the literature regarding the neural correlates of human navigation. On one side, a methodological review allows highlighting the main cognitive variables across fMRI studies; on the other side, a meta-analytic approach makes it possible to analyze the role of these variables by integrating data from several studies to identify brain areas that show a consistent response across studies and experimental variables.

The first step is to identify the main differences in the paradigms used in fMRI studies of human navigation. One of the most obvious differences is familiarity with the environment in which the participants have to navigate. In some studies, participants are presented with environments they have been very familiar with for years (Maguire et al. 1998; Nemmi et al. 2011). In other studies, they are exposed to the environment just before the experimental test (Berthoz 1997) or during the study (Iaria et al. 2007). In any case, successful navigation of both humans and animals depends on memories of the environment, that is, the degree of familiarity and the time lapse between the learning phase and recall. Montello (1998) reported that environments which are well known and familiar are more likely to be represented in a survey format (similar to cognitive maps) and that the format of representation, which influences the style of navigation, changes with degree of familiarity. Iaria et al. (2007) showed that learning a virtual environment activates different hippocampal areas than those activated during recall when learning is fully established. Furthermore, studies of other types of memory (i.e., episodic memory) have shown that areas involved in the recall of less familiar items (i.e., items that have been recently learned) are slightly different from those involved in the recall of very familiar items that have already been recalled several times (Henke 2010; Carr et al. 2011). Thus, it is very likely that the variations in brain activities observed in different studies of spatial navigation are linked to the different degree of familiarity (i.e. very familiar vs. recently learned) participants have with experimental environments. Another main difference between experimental paradigms is the type of environmental representation (egocentric vs. allocentric) elicited by the experimental task. When using egocentric representations, participants locate environmental items by referring to their own position (e.g., the door is on my left, the window is behind me on the right, etc.). Instead, in allocentric representation the position of the item is not linked to that of the participants (e.g., the door is northwest, the window is 5° south, etc.).

Once the main methodological axis in the spatial navigation literature has been identified, the second step (which is central to the current study) is to verify converging and consistent evidence in the current literature by means of a meta-analysis, integrating data from several studies. This will allow assessing the role of the main methodological variables of fMRI studies, overcoming the limitations of the single study approach, such as small sample size, low reliability and logical subtraction, which is sensitive only to differences between conditions.

First, we will review the fMRI studies within a theoretical framework that takes into account a) degree of familiarity with the environments and b) types of environmental representations and navigational strategies required by the experimental task. Then, we will carry out a meta-analysis on fMRI studies of human navigation using activation likelihood estimation (ALE) (Eickhoff et al. 2009) to verify involvement of the neural network identified in the review and differences related to the previously identified methodological variables.

The main aims of the meta-analysis were the following: (i) to find converging evidence of a specific and dedicated network for spatial navigation in the human brain, to overcome the discrepancies found in neuroimaging studies of human navigation; (ii) to test the hypothesis that well-learned, familiar environments and recently learned environments are processed by different neural substrates; and (iii) to assess the degree of overlap between the brain networks that mediate the egocentric and allocentric strategies employed in navigation.

## Paradigm: Navigating in Recently Learned and Familiar Environments

Neuroimaging studies of human navigation can be roughly divided into studies that focus on recently learned environments, that is, environments learned for experimental purposes (e.g. a university campus or a virtual environment) (Janzen and van Turennout 2004; Janzen and Weststeijn 2007; Janzen and Jansen 2010; Iaria et al. 2007, 2008, 2009; Schinazi and Epstein 2010) and studies that require participants to perform experimental tasks in familiar environments (Rosenbaum et al. 2004, 2007; Hirshhorn et al. 2011; Spiers and Maguire 2006; Ino et al. 2007). In the first type of paradigm (hereafter called the RL paradigm), the aim is to understand the neural basis of coding, storing and use of navigational knowledge. A good example of the RL paradigm is Iaria and colleagues’ study using the Cognitive Map Test (Iaria et al. 2007). These authors created a virtual environment that permitted good control over stimulation and allowed studying both learning and retrieval. The environment consisted of several buildings of different sizes and shapes and six clearly identifiable landmarks. The participants moved in the virtual environment by using a three-button keypad; each button corresponded to a different direction of movement. Latini-Corazzini et al. (2010) also used an RL environmental paradigm and developed a virtual reality task in which participants navigated to acquire the knowledge necessary to carry out the subsequent spatial tasks. In this case, the main environment was a small virtual town with a regular grid of streets and 21 buildings, most of which had no distinctive features. Nine buildings had specific signs (hotel, bank, etc.). In the practice phase participants could freely explore the town, but in the encoding phase they could only move along a defined route. Using an RL paradigm, Baumann et al. (2010) created a virtual “arena” consisting of an infinite plain. The arena contained four objects: three cylinders of different colors (landmarks) and one yellow pyramid (target). The pyramid had a virtual ‘light beacon’, which projected vertically from the apex and signaled the pyramid’s position when it was occluded by the landmarks. During the encoding phase, each participant had to navigate toward the target and remember its spatial position with respect to the three landmarks.

At variance with the RL paradigm, in some studies the environments used in the experimental tasks are already familiar to participants. In these studies (hereafter called the familiar (F) paradigm), the aim is to explore the recall and use of navigational knowledge. Spiers and Maguire’s (2007) “taxi-driver-task” is a very good example of an F paradigm. It presents novel tasks to participants who have gained thorough knowledge of the experimental environment over a long period of time. These authors used a virtual reproduction of London to assess the previously acquired spatial knowledge of taxi drivers, who had gotten their license by demonstrating good knowledge of the city. Rosenbaum and colleagues (Rosenbaum et al. 2004, 2007; Hirshhorn et al. 2011) carried out two studies involving mental navigation in the city of Toronto. Nemmi et al. (2011) asked participants who had lived in Rome for at least 5 years and had demonstrated good familiarity with the city centre to indicate whether three landmarks were shown in correct order along a route. Ino et al. (2002) tested participants who were very familiar with Kyoto City and during the fMRI asked them to mentally navigate from one place (named by the experimenter) to another.

It is noteworthy that in the only study which directly contrasted remote and recent learning of navigational information (Hirshhorn et al. 2011) the hippocampal formation was activated only for recently acquired spatial knowledge and the extra-hippocampal structures (i.e. parahippocampal cortex, lingual gyrus and precuneus) were engaged in the recall of remote spatial knowledge.

The debate over the role of increasing familiarity in both behavioral and neuroimaging data is related to the debate over the role of the hippocampus in declarative episodic memory and the contrasting findings in patients with medial temporal lobe (MTL) amnesia (Milner 2005; Bohbot and Corkin 2007). As in other forms of declarative memory, spatial memory seems to be compromised in patients with MTL lesions, but not all memories seem impaired to the same extent. Although it is widely recognized that patients with hippocampal and MTL damage cannot learn to navigate in a novel environment, there is evidence that they are usually able to navigate in environments learned before the damage (Habib and Sirigu 1987; Aguirre and D’Esposito 1999). This mirrors the well-known dissociation between new and old episodic memory in amnesic patients (Milner 2005), which is compatible with different longstanding theories about the hippocampal role: the declarative theory (Tulving 1987), the Standard model of memory consolidation (Squire and Alvarez 1995) and the multiple trace theory (Moscovitch et al. 2005; Moscovitch et al. 2006.). All of these models propose that some memories might survive hippocampal damage but they differ in the mechanism supposed to explain this survival. The declarative theory hypothesizes that the hippocampus is necessary only in the formation of episodic and spatial memories and that its role is time-limited because it is linked to the fixation of memories that will be stored in neo-cortical areas. The Standard model of memory consolidation proposed a gradual reorganization within long-term memory storage so that, as time passes after learning, the importance of the hippocampal formation gradually diminishes accordingly to the increasing of memory consolidation until a permanent memory trace develops which is independent from this region. By contrast, the multiple trace theory hypothesizes that a new trace is formed in the hippocampus every time a certain memory is recollected, so that older memories become more resistant to hippocampal damage or become semantic and independent from the hippocampus. According to the authors who support these theories, spatial memory and its failure following hippocampal damage is a valid model for studying the more general system of episodic memory.

The claim that old memories of familiar environments are retained in MTL-damaged patients who show impaired recall of recently acquired environments and are unable to learn totally new ones has been challenged (Nadel and Moscovitch 1997) and some studies have shown that hippocampal activity is not limited to the recall of recently learned environments (Niki and Luo 2002). Therefore, another aspect that remains controversial is the role of the hippocampus in remembering very familiar environments or spatial memories acquired in the distant past and often recollected. A study brining evidences in favor of the persisting of highly familiar spatial representation in spite of hippocampal damage is the one by Maguire et al. (2006), who showed that a taxi driver after extensive bilateral hippocampal damage retained the ability to navigate in a virtual reconstruction of the city of London. Since he was able to navigate by means of the main or principal routes but not using minor routes, the author concluded that a “sketch map” of an environment (i.e. a semantic type of spatial memory, opposed to an episodic one) could be remembered without hippocampal involvement (Maguire et al. 2006).

Anyway the relationship between hippocampus and familiarity of the spatial representations has been somewhat neglected in the functional literature and only a few studies have directly compared old and recent spatial memories (Niki and Luo 2002; Hirshhorn et al. 2011; Maguire et al. 2001) and have reported contrasting evidence. Starting from the above-described theoretical framework, a meta-analytic approach, assessing the consistence of the neural response across studies and experimental variables would clarify the role of the hippocampus and medial temporal lobe.

## Spatial Strategies: Egocentric and Allocentric Representations

Neuroimaging studies on spatial navigation can also be separated according to the type of representation participants have to retrieve to perform the task. As stated above, we chose to focus on egocentric (ego) and allocentric (allo) spatial representations.

Neuroimaging studies focusing on egocentric navigation can be divided into those assessing “offline” spatial egocentric memories and those assessing “online” egocentric-based navigation in real or virtual environments (Wolbers and Hegarty 2010). The first type of task requires participants to judge the order of two landmarks along a street (Rosenbaum et al. 2004), to recognize or recall landmark appearance (Janzen et al. 2007) or to judge whether a certain landmark was or was not along a certain route (Nemmi et al. 2011; Schinazi and Epstein 2010). The second type of task includes those requiring navigation along habitual routes in real familiar environments by means of mental imagery (Ino et al. 2002) or following trails or arrows along the route (i.e., so-called “route following”; Hartley et al. 2003).

By contrast, tasks assessing allocentric-based navigation are usually carried out in virtual environments in which participants have to navigate from a starting point to a goal in a condition that does not allow following the usual route because, for example, it is blocked (Iaria et al. 2007; Rosenbaum et al. 2004; Spiers and Maguire 2006). Although this hypothesis has been questioned (Maguire et al. 2006), it is believed that the presence of a blocked route forces participants to access the cognitive map of the environment to “find” a novel path (Iaria et al. 2007, 2008). Another way to force participants to use an allocentric representation is to ask them to “find” a shortcut between two locations so they will not repeat the route they have learned (Rosenbaum et al. 2004; Hirshhorn et al. 2011).

The above mentioned Cognitive Map Test (Iaria et al. 2007) is a paradigmatic allocentric task. It contains several crossroads and represents a city in which all buildings, except six landmarks, have the same texture. According to the authors, this task taps on the formation and use of a cognitive map, because participants are required to navigate from one landmark to another using the shortest path. Thus, to plan a novel path they have to recall a cognitive map. Iaria et al. (2007) found clear activation of the hippocampal formation: anterior regions were more active when participants were acquiring the allocentric representation of the virtual city and posterior regions were more active when they used the learned representation.

Novel learning of a new environment is not necessarily related to allocentric encoding. According to Montello (1998), the type of encoding and the specific representation recalled depend on the task requirements. Latini-Corazzini et al. (2010) presented a virtual environment similar to the one used by Iaria et al. (2007). After the learning phase, they asked participants to perform a “route” task and a “survey” task. In the route task, participants were required to follow a route and indicate which direction the path took at various crossing points. In the survey task, they had to indicate the direction in which a certain landmark (not immediately visible) was located with respect to a certain point on the route. To accomplish the first task, participants had to recall an egocentric representation of the route based on the direction of the path; to correctly perform the second task, they had to recall a survey representation of the environment by storing the geometrical configuration of each landmark.

To differentiate between egocentric and allocentric representations of the environment, Hartley et al. (2003) presented participants with two environments and asked them to learn one environment by following arrows that were visible along a path (egocentric task) and to learn the other one by freely exploring it (allocentric task). During the recall phase, participants accessed an egocentric representation in the first environment and an allocentric (presumably survey) representation in the second environment.

Maguire and colleagues (Maguire et al. 1998; Spiers and Maguire 2006) focused on tasks that required accessing the allocentric representation of known environments in their “taxi driver task”. In this task, participants had to take a passenger to a specific location, usually by avoiding some streets or having to change destination in the middle of the path. Being forced to change the habitual route should force participants to use a survey representation of the environment rather than an egocentric representation of habitual routes.

Egocentric and allocentric knowledge can also be tested by so-called mental navigation. In mental navigation tasks, participants have to use visual mental imagery. For example, to test both egocentric and allocentric representations Rosenbaum et al. (2004, 2007) and Hirshhorn et al. (2011) asked participants to mentally navigate in a very familiar environment. In tasks tapping egocentric representations, participants have to judge the correctness of a sequence of landmarks along a route to determine which of two landmarks is closest to them or mentally navigate a familiar route while naming the streets encountered along the path. In tasks tapping allocentric representations, participants have to judge which of two landmarks is closest to a third landmark specified in the instructions or to mentally navigate along blocked routes naming all the streets crossed by the path.

From a neural point of view, many studies have shown that egocentric navigation is subserved by an ensemble of areas related to landmark knowledge (i.e., parahippocampal place area, Epstein and Ward 2010), egocentric spatial representation in the parietal cortex (i.e., precuneus and cuneus, inferior parietal lobe) and heading vectors possibly coded by head-direction cells in the retrosplenial cortex. Instead, allocentric navigation seems mainly related to the hippocampal cortex (Tolman 1948; O’Keefe and Nadel 1978; Maguire et al. 1998) and, more specifically, to a network of areas containing place cells (hippocampus) and grid cells (entorhinal cortex). Additional hypothetical cells, namely boundary vector cells and head-direction cells, which should be located in the retrosplenial complex, have been included in models of human navigation, such as in the BBB model (Byrne and Becker 2007). It is believed that these structures, and the cell populations within them, interact to update the participant’s current position in space by calculating the heading vector towards the navigational goal and planning the shortest or most feasible route to reach the goal (Byrne and Becker 2007). Head-direction cells were found in the cortex of rodents (Taube et al. 1990) and areas coding for head direction have been described in parietal lobe in humans in some papers (see for example, Schindler and Bartels 2013). However, a clear localization of neural correlates of head direction cells in humans as well as the understanding of their role in human navigation are still matter of debate, also due to the fact that the impossibility to move the heads during fMRI scan significantly reduces the possibility to test the head-direction system.

Neuroimaging studies have repeatedly shown activations in the hippocampal formation, parietal cortex and retrosplenial regions during tasks involving both egocentric (Wolber et al. 2004; Latini-Corazzini et al. 2010; Galati et al. 2000) and allocentric (Latini-Corazzini et al. 2010; Iaria et al. 2007) features. While these results suggest that all of these areas are involved in navigation, their specific roles have not yet been identified. In this light, a meta-analytic approach, within the theoretical framework above described, could be useful to identify brain areas that show a consistent response across different studies and experimental settings. Indeed, the differences in cerebral activations we found in the studies cited in the present review of literature could derive from differences in paradigms and tasks used. It is important to note that in the context of fMRI, an experimental task is a way to elicit and to observe a particular cognitive process and that different experimental tasks, coupled with a well-studied control task, can be used to observe the same cognitive process. A great variance in localization and extension of the clusters of cerebral activation found in studies focusing on the same cognitive process (i.e. egocentric or allocentric navigation) or on the same type of spatial representation (i.e. egocentric or allocentric spatial representation), despite the difference in the task used, is certainly surprising and need a deeper analysis. A meta-analysis of fMRI studies, going beyond the limitations of a single study approach, could be a way to fill this gap.

## Meta-Analysis

### Inclusion Criteria for Papers

The database search on PubMed was performed using the following string: “fmri AND (navigation OR egocentric OR allocentric OR map) NOT gene NOT genetic NOT DNA NOT heart NOT patients NOT cellular NOT social NOT psychopathy”. A total of 42 studies were found.

Our a-priori inclusion criteria for papers were: 1) Inclusion of whole-brain analysis performed using functional magnetic resonance imaging (fMRI); thus, we excluded positron emission tomography (PET) studies, electrophysiology studies and papers that reported only results from ROI analysis. 2) Provision of coordinates of activation foci, either in Montreal Neurological Institute (MNI) or Talairach reference space. 3) All participants in the studies had to be young and healthy. Studies that included healthy elderly adults were excluded to avoid the effects of aging on navigation. 4) All neuroimaging studies had to include a visuo-perceptual control condition to exclude all activations that were not directly connected to navigation. 5) The experimental tasks required participants to recall a learned environment. They had to make a decision about the pathway learned before or to reach a position in the space by pressing keys or using a joystick. The space was either a virtual reality or a real environment and the task required either allocentric or egocentric strategies. Studies that did not focus on spatial navigation were excluded from the meta-analysis. 6) Only group studies were included. 7) There could be no pharmacological manipulation.

Using these criteria, we selected 24 studies. Meta-analysis was carried out on 66 neuroimaging experiments (described in the 24 published studies) using the “activation likelihood estimation” (ALE) analysis. A total of 1023 participants participated in these trials. Studies are summarized in Tables 1 and 2.

**Table 1:** Familiar and recently learned environment

Experimental paradigm^a^	Paper^b^	Subjects^c^	Experiments^d^
Familiar environment			
	Hirshhorn et al. 2011	13	3
	Ino et al. 2002	16	1
	Nemmi et al. 2011	19	1
	Rosenbaum et al. 2004	10	5
	Rosenbaum et al. 2007	7	8
	Schinazi and Epstein 2010	16	1
	Spiers and Maguire 2006	20	7
Novel environment			
	Baumann et al. 2010	17	2
	Brown et al. 2010	22	2
	Burgess et al. 2001	13	3
	Gron et al. 2000	24	1
	Hartley et al. 2003	16	2
	Iaria et al. 2007	9	1
	Iaria et al. 2008	10	4
	Janzen and Jansen 2010	20	3
	Janzen et al. 2007	15	2
	Jordan et al. 2003	10	2
	Latini-Corazzini et al. 2010	16	2
	Ohnishi et al. 2006	56	1
	Rauchs et al. 2008	16	4
	Schinazi and Epstein 2010	16	2
	Viard et al. 2011	18	1
	Wolbers 2005	17	1
	Wolbers et al. 2007	16	1
	Xu et al. 2010	20	6

^a^Experimental paradigm used in each study

^b^References to studies

^c^Number of subjects included in each study

^d^Total number of experiments in each paper

**Table 2:** Allocentric and egocentric studies

Navigational strategy^a^	Paper^b^	Subjects^c^	Experiments^d^
Allocentric			
	Hartley et al. 2003	16	2
	Hirshhorn et al. 2011	13	3
	Iaria et al. 2007	9	1
	Jordan et al. 2003	10	1
	Latini-Corazzini et al. 2010	16	1
	Ohnishi et al. 2006	56	1
	Rauchs et al. 2008	16	4
	Rosenbaum et al. 2004	10	3
	Rosenbaum et al. 2007	7	4
	Spiers and Maguire 2006	20	6
	Wolbers 2005	17	1
	Xu et al. 2010	20	3
Egocentric			
	Baumann et al. 2010	17	2
	Brown et al. 2010	22	1
	Burgess et al. 2001	13	3
	Gron et al. 2000	24	1
	Iaria et al. 2008	10	4
	Ino et al. 2002	16	1
	Janzen and Jansen 2010	20	3
	Janzen et al. 2007	15	2
	Latini-Corazzini et al. 2010	16	1
	Nemmi et al. 2011	19	1
	Rosenbaum et al. 2004	10	2
	Rosenbaum et al. 2007	7	4
	Schinazi and Epstein 2010	16	3
	Spiers and Maguire 2006	20	1
	Viard et al. 2011	18	1
	Wolbers et al. 2007	16	2
	Xu et al. 2010	20	2

^a^Navigational strategies required in reported studies

^b^References to studies

^c^Number of subjects included in each study

^d^Total number of experiments in each paper

### Activation Likelihood Estimation

Activation likehood estimation (ALE) analyzes the probability that a voxel will contain at least one of the activation foci; it is calculated at each voxel and results in a thresholded ALE map. In other words, ALE assesses the overlap between foci by modelling the probability distributions centered at the coordinates of each one (Eickhoff et al. 2009).

A general ALE meta-analysis was performed on the foci derived from the selected studies on navigation (Tables 1 and 2). The coordinates of the foci were taken from original papers. A total of 782 foci were reported in 66 experiments including 1023 participants.

We also performed four separate ALE analyses on four categories of studies in relation to the type of familiarity paradigm (recently learned vs. familiar environment) and spatial strategies (egocentric vs. allocentric strategies) used in the experiment.

Regarding the categorization of studies according to degree of familiarity, we separated experiments according to whether the environment used in the study was unknown to the participants before they were recruited for the study (recently learned environment paradigm, RL) or was already known before they were recruited (familiar environment paradigm, F), for example, their home town (Maguire et al. 1998; Nemmi et al. 2011), college campus (Epstein and Ward 2010) or a specific district (Hirshhorn et al. 2011). We categorized 40 experiments as RL environments and 26 as F environments.

Regarding spatial strategies, the allocentric strategy category included studies that required participants to access a cognitive map of the environment or tasks that forced them to rely on a survey representation of the environment (e.g., to find a shortcut in a blocked-route task). In the egocentric strategy category, we included studies in which participants had to access route knowledge of the environment and in which tasks tapped offline egocentric knowledge of an environment, by means of a landmark-based or a route-following strategy (e.g., participants had to judge the relative distance between landmarks and their own position, Rosenbaum et al. 2007). As the authors never stated whether the tasks were egocentric (ego) or allocentric (allo), two experimenters (F.N. and M.B.) independently classified the studies. They classified all but one study (in Xu et al. 2010) in the same category. The data from this study were included in the general analysis and in the individual ALE analysis of the paradigm (RL vs. F environment) but not in the analysis of the neural substrate of navigational strategies. A total of 30 experiments were defined as allocentric and 34 as egocentric (see Tables 1 and 2 for more details).

After carrying out separate ALE analyses on the categories of studies [*paradigm* (recently learned vs. familiar environment) and *spatial strategies* (egocentric vs. allocentric strategies)], we performed two contrast analyses to directly compare the effects of the paradigms [(F > RL) and (RL > F)] and strategies [(allo > ego) and (ego > allo)]. These contrast analyses allowed highlighting voxels whose signal was greater in the first than the second condition. We also carried out a conjunction analysis of *paradigms* [(RL)^(F)] and *strategies* [(allo)^(ego)] to identify voxels that subtended both paradigm and strategy conditions.

The ALE meta-analysis was performed using GingerALE 2.1.1 (brainmap.org) with MNI coordinates (Talairach coordinates were automatically converted into MNI coordinates by GingerALE.). According to Eickhoff et al.’s (2009) modified procedure, the ALE values of each voxel in the brain were computed and a test was performed to determine the null distribution of the ALE statistic of each voxel. The FWHM value was automatically computed, because this parameter is empirically determined (Eickhoff et al. 2009). The thresholded ALE map was computed using p values from the previous step and a False Discovery Rate (FDR) at the 0.05 level of significance (Tom Nichol’s FDR algorithm). Moreover, a minimum cluster size of 200 mm^3^ was chosen. A cluster analysis was performed on the thresholded map. The ALE results were registered on an MNI-normalized template (brainmap.org) using Mango (www.ric.uthscsa.edu/mango).

## Results

### General Meta-Analysis

**Fig. 1:**
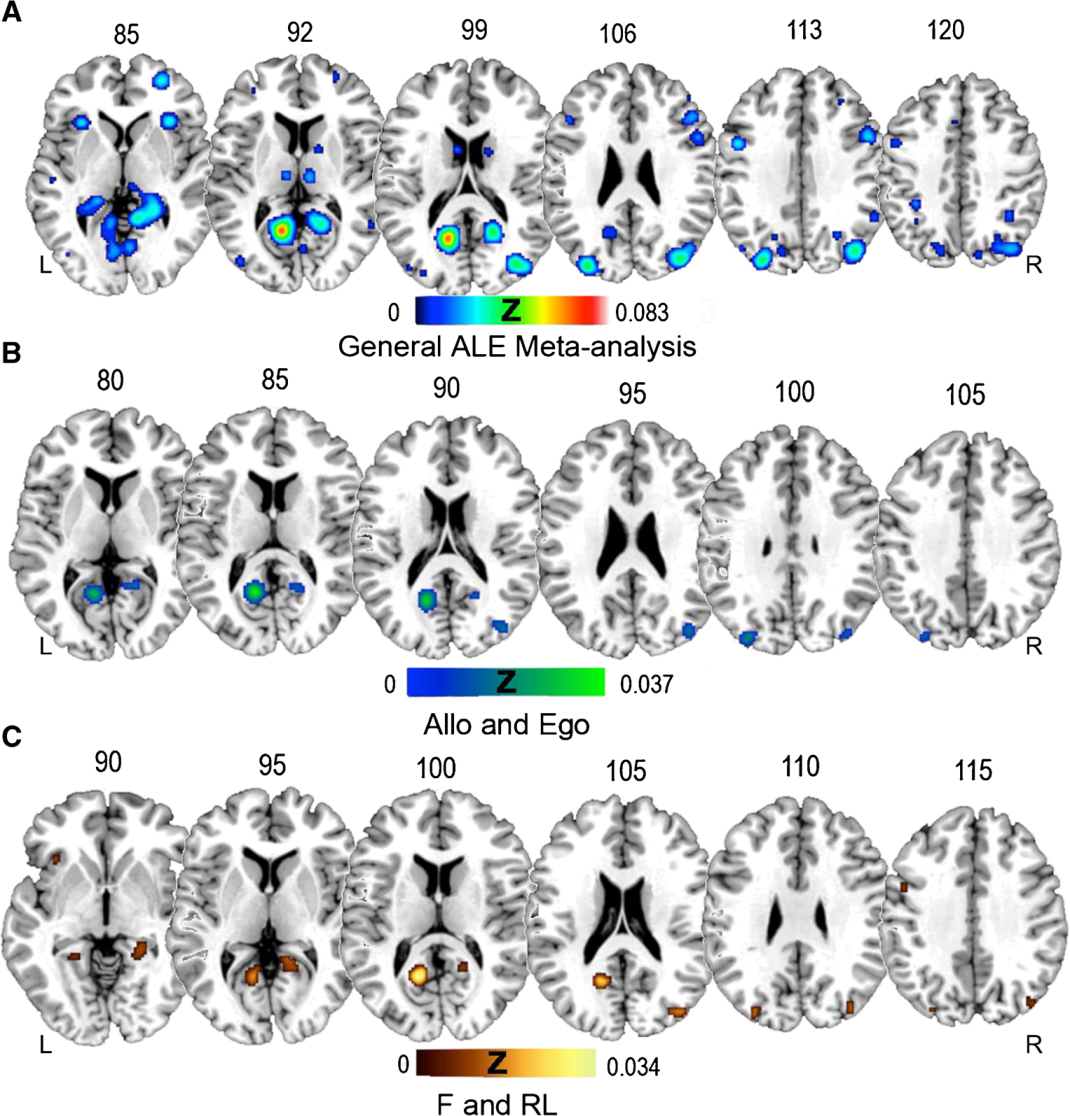
**a** Results of general ALE meta-analysis: a widespread network of areas seems to subtend the human ability to orient navigation. This network includes the medial temporal lobe, parietal and occipital areas, as well as the cerebellum and frontal lobe. **b** Areas showing activation in both egocentric and allocentric spatial strategies span from the occipital to the frontal lobe, as revealed by conjunction analysis egocentric [AND] allocentric strategies. **c** Areas showing activation for both familiar and recently learned environments, as revealed by conjunction analysis F [AND] RL environments

In the general ALE analysis, we found 25 clusters in both the right and left hemispheres (Fig. 1a) (Detailed information about cluster are reported in supplementary materials, table S1). Some of these clusters extended from the right to the left hemisphere and vice versa (e.g. clusters 2, 5 and 11). Others were localized in one of the two hemispheres (clusters 1, 3, 4, 14 and 17). In the right hemisphere, cluster 1 extended from the parahippocampal gyrus to the posterior cingulate cortex and the anterior cerebellum. In the left hemisphere, cluster 3 extended from the parahippocampal gyrus to the anterior cerebellum. An extensive area of ALE peak was found in the left and right precuneus (clusters 4, 5, 17, 25); in the right hemisphere, the precuneus was strongly related to the ALE peaks in the superior and middle occipital gyrus (cluster 4), and in the left hemisphere, to the left superior parietal lobe and the right precuneus (cluster 5). We also found clusters in frontal areas, specifically, the medial frontal gyrus, middle frontal gyrus, inferior frontal gyrus, and precentral gyrus (clusters 6, 7, 10, 15, 16, 18). The superior parietal lobe showed ALE peaks together with the inferior parietal lobe (cluster 14) (ALE peaks were found in the superior parietal lobe and the inferior parietal lobe (cluster 14)) in the left hemisphere, and the supramarginal gyrus was active on the right side (cluster 23). Clusters in the occipital cortex extended from the lingual gyrus to the posterior cingulate cortex on the left (cluster 2). We found a cluster that included the middle occipital gyrus and the superior occipital gyrus (cluster 4) in the right hemisphere, but only the middle occipital gyrus in the left hemisphere (clusters 8 and 21). Finally, another cluster in the left hemisphere included the cuneus and the precuneus (cluster 17). We also found a cluster in the right caudate nucleus (cluster 20).

Results of this general ALE analysis, which showed involvement of the parahippocampal cortex, precuneus and lingual gyrus (Rosenbaum et al. 2004; Wegman and Janzen 2011; Schinazi and Epstein 2010; Epstein 2008), are in complete agreement with the literature on navigational processes and their neural correlates. We also found consistent and extended clusters in the parietal cortex and frontal areas, which highlights the importance of these structures in navigation.

### Paradigm

#### Familiar Environment

In the ALE analysis of the F environment studies, the environment, used as stimulus, was learned through natural exploration (e.g., during daily life activities) and not for experimental purposes. This type of learning also implies that the environmental knowledge used for the experiment was acquired a long time before the study and with no restrictions concerning learning time and modalities (i.e., verbal instructions, free exploration, use of paper maps and/or combinations of modalities). We found a large cluster extending from the right parahippocampal formation to the right posterior cingulate cortex. Activations of both of these structures were also present in the left hemisphere. An occipital cluster extending from the superior to the middle gyrus was active in the right hemisphere. Other clusters in the right hemisphere included the superior temporal gyrus, cingulate gyrus, precuneus, middle frontal gyrus and anterior cingulate cortex. In the left hemisphere, we found foci in the middle and inferior frontal gyrus, superior temporal gyrus, superior occipital gyrus and inferior parietal lobule (see table S2 in supplementary materials for more details).

#### Recently Learned Environments

The ALE meta-analysis of RL environment studies, that is, of studies involving real or virtual environments learned ad hoc for research purposes shown principal clusters in the parahippocampal formation in the right hemisphere, the precuneus bilaterally and the left superior parietal lobule. In the left hemisphere, a cluster was found in the parahippocampal formation and the hippocampus. This is relevant to the animated debate over the role of the hippocampus in navigation. Presumably, the hippocampus is mainly related to acquisition and/or recall of a recently learned space, created ad hoc for the study, and less to a spatial task requiring access to long-term knowledge of familiar, naturally acquired environments.

We found a cluster in the superior parietal lobule bilaterally and the inferior parietal lobule of the left hemisphere. Foci were also found in the precuneus bilaterally and the left cuneus. The middle occipital gyrus was bilaterally activated, whereas the superior occipital gyrus was activated only in the left hemisphere and the lingual gyrus only in the right hemisphere. In the left hemisphere, we also found foci in the posterior cingulate cortex. The inferior frontal gyri were bilaterally activated, whereas the superior frontal and middle frontal gyri were activated in the right hemisphere and the medial frontal gyrus in the left hemisphere (see table S3 in supplementary materials for more details).

#### Contrast Analysis

##### Familiar vs. Recently Learned Environment

**Fig. 2:**
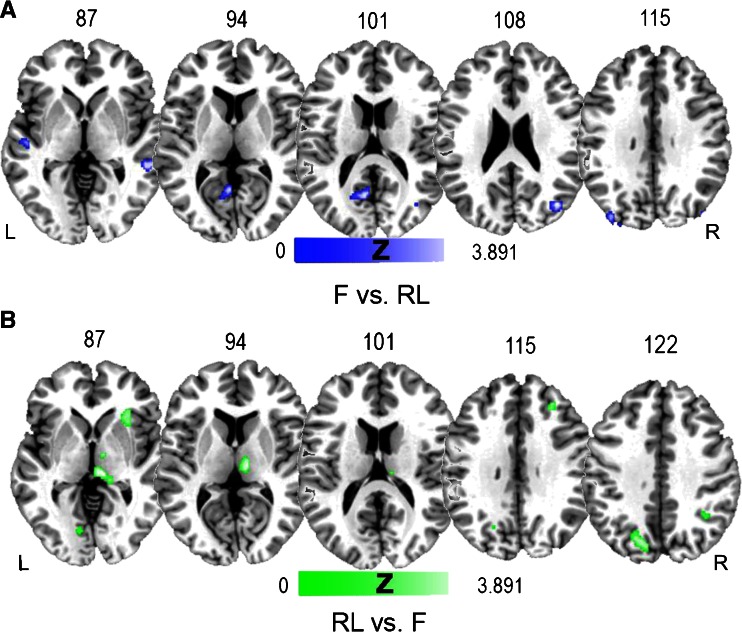
**a** Areas showing higher activation for familiar environments than recently learned ones, as revealed in the contrast between F vs. RL environments. This network of areas includes a cluster in the middle temporal gyrus in the right and posterior cingulate cortex, middle frontal gyrus and superior temporal gyrus of the left hemisphere. **b** Areas showing higher activation of RL than F environments, as revealed by the contrast between RL vs. F environments. This network includes the right parahippocampal gyrus, precuneus, insula and inferior parietal lobule, left cuneus, precuneus and lingual gyrus

Results of the T contrast [F > RL] showed clusters of voxels that were more activated by a familiar environment in both left and right hemispheres (Fig. 2a). We found a cluster in the middle temporal gyrus on the right and foci in the posterior cingulate cortex, middle frontal gyrus and superior temporal gyrus on the left.

These results suggest the existence of a specific network of cerebral areas and specific cognitive processes for well-learned familiar environments (Hirshhorn et al. 2011).

##### Recently Learned vs. Familiar Environment

The opposite T contrast [RL > F] showed clusters of voxels that were more activated by RL (Fig. 2b). We found clusters bilaterally distributed, including the precuneus and cuneus in the left hemisphere and the precuneus, insula, inferior parietal lobule and parahippocampal gyrus in the right hemisphere.

#### Conjunction Analysis

Conjunction analysis [RL ^ F] showed that the two types of paradigms partially share a neural network consisting of the fusiform, lingual and middle occipital gyri bilaterally and the calcarine cortex and middle frontal gyrus in the left hemisphere (Fig. 1c).

### Spatial Strategies

#### Allocentric

The ALE meta-analysis performed on studies that used tasks which rely on allocentric strategies showed clusters (see table S4 in supplementary materials for more details) mainly localized in the parahippocampal gyrus in both right and left hemispheres. Furthermore, bilateral foci were found in the precuneus and lingual gyrus as well as the middle temporal and middle occipital gyri in the right hemisphere and the superior temporal gyrus in the left hemisphere. Other bilateral clusters were found in the frontal cortex.

#### Egocentric

In the right hemisphere, results of the ALE meta-analysis of studies whose tasks relied on egocentric strategies (see table S5 in supplementary materials for more details) showed the presence of a cluster involving the parahippocampal gyrus, cerebellum and posterior cingulate cortex, and a cluster that extended from the parahippocampal gyrus to the amygdala. Clusters were also found in the parahippocampal gyrus and posterior cingulate cortex of the left hemisphere, the precuneus in both hemispheres and the superior and middle occipital gyrus, middle frontal gyrus and superior frontal gyrus of the right hemisphere. In the left hemisphere, we found foci in the middle frontal gyrus, superior occipital gyrus, cuneus and precuneus, medial frontal gyrus, lingual gyrus, superior parietal lobule and inferior occipital gyrus. Egocentric strategies also seemed related to activation in the right caudate nucleus, a structure shown to be related to egocentric navigational tasks (Latini-Corazzini et al. 2010), as well as to a cluster in the superior parietal lobule, which is strongly related to egocentric strategies (Latini-Corazzini et al. 2010; Shelton and Gabrieli 2002).

Our finding of involvement of the amygdala as well as the parahippocampal formation in the right hemisphere seems very interesting and somewhat unexpected.

Once again, results highlighted the role of the parahippocampal formation bilaterally, the lingual gyrus and the parietal lobe (i.e. the precuneus).

Results of contrast analysis showed differences between the two strategies.

#### Contrast Analysis

##### Allocentric vs. Egocentric

No suprathreshold clusters were revealed by T contrast [allo > ego], suggesting that the neural areas involved in the two types of navigational strategies partially overlap. This is at odds with results of previous studies that directly compared egocentric and allocentric representations (Galati et al. 2000; Committeri et al. 2004) but confirms what already partially emerged in studies that directly compared survey and route encoding of spatial information (Shelton and Gabrieli 2002).

##### Egocentric vs. Allocentric

**Fig. 3:**
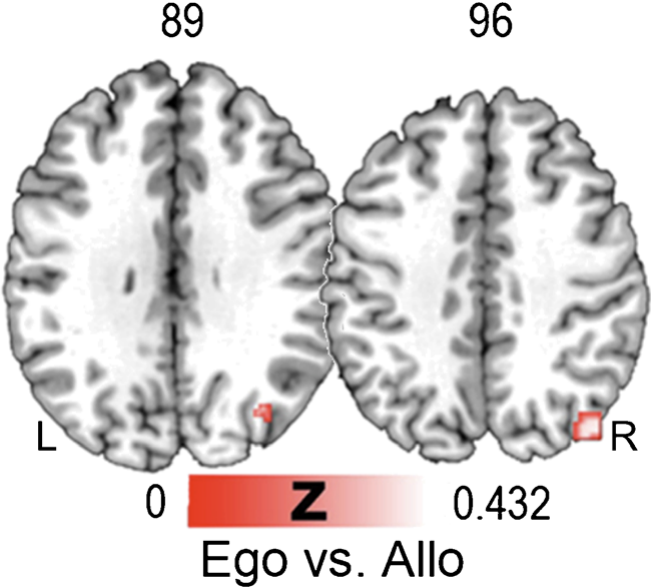
Areas showing higher activation for egocentric than allocentric strategies, as revealed by the contrast between ego vs. allo strategies. A parieto-occipital network that includes the right superior occipital gyrus, angular gyrus and precuneus subtends egocentric representation of space

At variance with the previous analysis, T contrast [ego > allo] strategies showed an extended cluster that included the superior occipital gyrus, angular gyrus and precuneus in the right hemisphere (Fig. 3).

#### Conjunction Analysis

Conjunction analysis [allo ^ ego] showed that the two spatial representations share a common network including the fusiform gyrus, insula, lingual gyrus, precuneus, cuneus, and superior frontal lobe bilaterally. Moreover, there was an overlap in the right middle occipital gyrus, left precentral gyrus and left middle frontal gyrus (Fig. 1b).

## Discussion

**Fig. 4:**
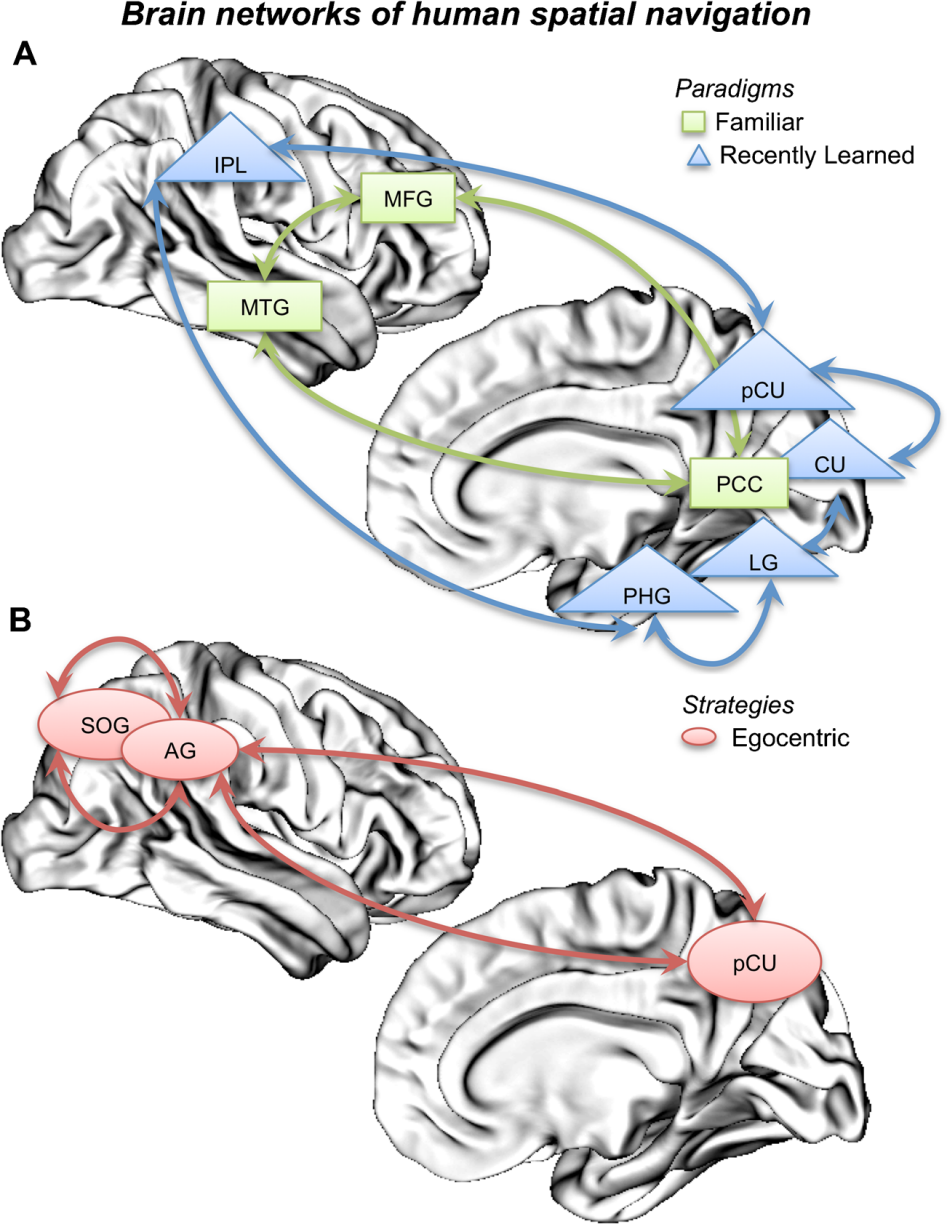
**a** Diagram shows the proposed network of human spatial navigation, as revealed by contrast analysis of paradigms (F vs. RL and RL vs. F). *Green rectangle* shows the subset of areas of navigation across F environments (*MFG* middle frontal gyrus, *MTG* middle temporal gyrus, *PCC* posterior cingulate cortex). *Blue triangle* shows the subset of areas involved in processing RL environments (*IPL* inferior parietal lobule, *pCU* precuneus, *CU* cuneus, *LG* lingual gyrus, PHG parahippocampal gyrus). **b** Diagram shows the proposed network of human spatial navigation, as revealed by contrast analysis of strategies (ego vs. allo). *Red circle* shows the subset of areas of egocentric representation of space (*SOG* superior occipital gyrus, *AG* angular gyrus, *pCU* precuneus)

This ALE meta-analysis clarified some important issues. First, our finding of converging evidence of a specific and dedicated network for spatial navigation in the human brain (Fig. 4) explains some of the discrepancies in neuroimaging studies and corroborates models of human navigation (Byrne and Becker 2007; Kravitz et al. 2011; Chrastil 2013). Second, going beyond the limitations of the single study approach, our results strongly support the hypothesis that there are different neural substrates for navigating in a well-learned, familiar environment and a recently learned environment. Finally, this analysis helps clarify the extent of the overlap between the brain networks of the egocentric and allocentric strategies employed in navigation (see table S6 in supplementary materials for more details).

The results of the *general ALE meta-analysis* emphasize the role of the parahippocampal gyrus and retrosplenial cortex in navigation. Previously, both of these areas were often associated with navigational processes (Epstein 2008; Vann et al. 2009) and were hypothesized to play separate and complementary roles in human navigation (Epstein et al. 2007; Iaria et al. 2007), particularly in the retrieval and localization of visual scenes (Epstein 2008; Hirshhorn et al. 2011). Our findings also confirm involvement of the parietal lobes (Sack 2009) in human navigation as well as the middle occipital gyri bilaterally (Epstein et al. 2007; Rosenbaum et al. 2004) and the caudate nucleus. The importance of the frontal areas in human navigation was confirmed by bilateral activations in the middle frontal gyri, as evidenced by the general ALE. As most studies included in the meta-analysis (Rosenbaum et al. 2004; Spiers and Maguire 2006; Ekstrom and Bookheimer 2008) required that participants “find a way” to perform a navigational problem-solving task, we suggest that the frontal areas may have a significant role in planning navigation, especially when detours are required. As few studies have investigated this point, it is still unclear whether navigational planning and problem solving differ from other types of planning and problem solving from cognitive and neural perspectives. We also observed cerebellar activations, which need to be further investigated.

### Paradigms: Recently Learned and Familiar Environments

The contrast between studies using paradigms of familiar environments and recently learned environments resulted in significant differences in both directions. A fronto-temporal-parietal network (including the middle frontal gyrus, posterior cingulate cortex and superior temporal gyrus) seems to be involved in processing F environments (familiar vs. recently learned environments, Fig. 2a), whereas activations of the parahippocampal formation, lingual gyrus and fusiform regions are evidenced by RL environment paradigms (recently learned vs. familiar environments, Fig. 2b). Hirshhorn et al. (2011) observed that degree of familiarity affects the networks involved in navigational tasks. This is also consistent with findings reported in the neuropsychological literature that lesions in the parahippocampal cortex cause anterograde disorientation (i.e., the inability to learn novel routes and create representations of novel environments) but do not affect the ability to orient and navigate in familiar environments, thus sparing spatial knowledge acquired before the lesion (Habib and Sirigu 1987; Aguirre and D’Esposito 1999). This result is also consistent with the Standard model of memory consolidation (Squire and Alvarez 1995), which posited a time-limited role of the hippocampus for declarative memories.

However, we cannot exclude that the possible role of the hippocampal formation in recalling a recently learned environment is consistent with its proposed role in novelty detection and orienting reactions (Vinogradova 2001; Kumaran and Maguire 2005, 2006, 2007). Indeed, according to the multiple trace theory recently acquired environments (at variance with familiar environments) may require further consolidation of memory traces by means of hippocampal activations. Also, as the RL environments are not yet completely consolidated, they may still make use of the hippocampus as a comparator, similar to novel environments when they are being acquired for the first time.

As to familiar environment paradigms, lesions in the posterior cingulate cortex, which is part of the fronto-temporo-parietal network identified in familiar environment paradigms, result in deficits in orienting and navigating in environments that were familiar before the lesion (Aguirre and D’Esposito 1999). Areas involved in the network that processes familiar environments are also strongly related to egocentric spatial representations (Galati et al. 2000) and the translation of representations from allocentric to egocentric formats and vice versa (Byrne and Becker 2007). Thus, lesions in these areas may affect the recall of knowledge about familiar environments and prevent its transformation from an allocentric format stored in long-term memory (Montello 1998) to an egocentric format used for driving actual navigation (Byrne and Becker 2007).

In conclusion, these results suggest that recently learned and familiar environments are processed by recruiting partially different networks. The first network includes the parahippocampal, fusiform and lingual gyri and is involved in processing memories relative to recently learned environments. The second network includes the middle frontal gyrus, posterior cingulate cortex and superior temporal gyrus and is involved in recalling familiar environments.

### Spatial Strategies: Allocentric and Egocentric Representations

The ALE analysis of navigational strategies showed that different and only partially overlapping systems are involved in processing allocentric and egocentric strategies. Conjunction analysis between allocentric and egocentric strategies demonstrated that they share a common network of areas (i.e., fusiform gyrus, insula, lingual gyrus, precuneus, cuneus, superior frontal lobe bilaterally, right middle occipital gyrus, left precentral gyrus and middle frontal gyrus).

Interestingly, the individual ALE on allocentric strategies revealed a cluster in the left superior temporal gyrus. The role of this structure in allocentric strategies is consistent with the hypothesis that it contributes to the formation and use of allocentric representations through the processing of categorical spatial relations (van Asselen et al. 2008). In any case, the contrast between allocentric and egocentric studies failed to show any suprathreshold cluster, demonstrating that allocentric encoding recruits a subset of areas also by egocentric encoding, in agreement with Shelton and Gabrieli (2002).

Regarding egocentric strategies, the ALE analysis of egocentric vs. allocentric strategies (Fig. 3) showed activation in the right precuneus and angular gyrus. This finding confirms the existence of a dedicated network for the egocentric representation of space in the right hemisphere, including areas in the parietal cortex (probably related to spatial representation) and the retrosplenial cortex (possibly coding heading vectors by means of head direction cells).

Interestingly, patients with right brain damage often have a deficit in spatial navigation and wayfinding (Aguirre and D’Esposito 1999) and lesions of the right precuneus and angular gyrus lead to egocentric disorientation (Aguirre and D’Esposito 1999). Several studies also suggest that the retrosplenial cortex is involved in egocentric spatial navigation and that its lesioning may lead to a condition called Heading Disorientation (Aguirre and D’Esposito 1999; Takahashi et al. 1997) or Retrosplenial Amnesia (Rudge and Warrington 1991). This network is probably also involved in translating allocentric representations of space into egocentric ones and vice versa (Byrne and Becker 2007).

Some caution is required in interpreting the results of comparison between egocentric and allocentric strategies. First of all, in discussing the differences between allocentric and egocentric strategies, it has to be taken into account that fMRI studies of egocentric navigation are intrinsically limited by the nature of the neuroimaging technique. Indeed, ecological egocentric navigation, especially in animal models, is supposed to heavily depend on internally generated cues, such as idiothetic cues (for example, proprioceptive, vestibular, optic flow inputs). In this light, the mandatory absence of actual motion in fMRI, excluding the presence of any idiothetic cue, affects the egocentric-based processing. This pervasive limitation across fMRI studies of egocentric navigation could account at least in part of the similarity between allocentric and egocentric strategies that resulted from our meta-analysis. Secondly, the *a posteriori* assignment of the studies to the egocentric and the allocentric navigational strategies could have weakened the differences in the meta-analysis results due to a mis-classification of studies or the overlapping in the strategies that may be used in performing some tasks. Indeed, the authors did not always explicitly report the kind of strategy their study aimed to analyze. However, in all of the studies the type of strategy the authors had sought for their tasks can be easily detected, even when it is not explicitly described in introduction, by the description of tasks themselves and by the discussion, where authors tried to link their functional findings to specific cognitive processes. Moreover, in most of the studies included in the meta-analysis, authors elicited a specific strategy by adopting paradigmatic tasks specifically developed to tap just a definite strategy rather than explicitly instructing subjects to follow that definite strategy. Thus it is possible that, despite the author’s intention to evaluate the neural bases of a navigational strategy (for example egocentric strategy) by means of a paradigmatic task (for example, a route-following task), actually some subjects perform the task by relying on the other strategy (for example, by relying on an allocentric strategy). It should, however, be consider that this is a common problem in cognitive neuroscience, since we can never be completely sure that subjects perform any experimental task by relying on the strategy authors meant to test. In any case, even being cautious, we retain that present results offer import suggestions for understanding the complex human navigational system and also suggest directions for future studies, which should pay attention in the more clearly defining the strategy analyzed and also in contrasting different strategies in the same study.

## Conclusions and Future Directions

Our meta-analysis confirms current models of human navigation, which propose that navigational memory and navigation itself are achieved by means of multi-process systems involving a widespread set of neural areas (Fig. 4) (Byrne and Becker 2007; Montello 1998; Siegel and White 1975; Brunsdon et al. 2007; Chrastil 2013). This meta-analysis allowed us to identify clusters of areas that form specific functional networks, which are selectively involved in different processes. The first differentiation concerns degree of familiarity with the environment. Our data suggest the following: the parahippocampal gyrus and fusiform and lingual gyri are part of a neural system that codes and stores environmental information; the middle frontal gyrus, posterior cingulate cortex and superior temporal gyrus are responsible for the recall of stored environmental information; the posterior cingulate cortex is more involved in transforming information from the allocentric format, in which it is stored in long-term memory (Byrne and Becker 2007), into the egocentric format necessary for driving actual navigation; and the frontal areas are more involved in planning routes in the recalled environments. Partially different networks are also involved in processing allocentric and egocentric representations, which are the core of different navigational strategies. Our results show that, although allocentric representation recruited a subset of areas also involved in egocentric representations, the latter are achieved by means of a specific network including includes the right precuneus and the angular gyrus. Allocentric representations rely on activations of the left superior temporal gyrus, a structure that is also involved in categorical spatial relations and perhaps also in so-called “spatial language”.

Finally, we wish to briefly discuss the involvement of two sets of cerebral areas, revealed by the present ALE, whose roles have been little investigated in human navigation. First, the results revealed a set of frontal areas, which could have a significant role in planning navigation. At the moment, however, it is unclear whether navigational planning is processed by the same systems involved in other types of planning or by specific subsets of frontal areas. Second, the analysis suggests that the cerebellum may have an important role in spatial navigation. But, due to the lack of studies on this issue the specific role of the cerebellum is still unclear. Experimental studies in rodents showed specific reduction of efficiency during navigational tasks performed by cerebellar-damaged rats (Foti et al. 2009; review in Petrosini et al. 1998) as did neuropsychological studies with patients (Molinari et al. 2004; Schmahmann 2004). In any case, to our knowledge the role of the cerebellum in human navigation has never been analyzed in detail. Thus, future studies specifically aimed at assessing the role of this region are necessary in order to understand the cerebellar contribution to navigational processes.

Both of these findings suggest the need for further studies aimed at investigating the role of areas other than those currently considered to be involved in human navigation.

## Electronic supplementary material

Below is the link to the electronic supplementary material.

Table S1 Results of general ALE meta-analysis (PDF 25 kb)

Table S2 Results of ALE meta-analysis on familiar environments (PDF 22 kb)

Table S3 Results of ALE meta-analysis on recently learned environments (PDF 25 kb)

Table S4 Results of ALE meta-analysis on Allocentric studies (PDF 21 kb)

Table S5 Results of ALE meta-analysis on Egocentric studies (PDF 23 kb)

Table S6 Contrast analysis between different strategies (Allocentric *vs.* Egocentric) and different paradigms (Recently learned *vs.* Familiar) (PDF 54.7 kb)
