# Supplementary material for: Neuropsychology of Environmental Navigation in Humans: Review and Meta-Analysis of fMRI Studies in Healthy Participants
Source: Neuropsychol Rev. 2014 Feb 1;24(2):236–51. doi: 10.1007/s11065-014-9247-8 (PMC4010721; doi:10.1007/s11065-014-9247-8)
Supplement: Supplementary file 1 — Results of general ALE meta-analysis (PDF 25 kb) [file 11065_2014_9247_MOESM1_ESM.pdf]

Table S1. Results of general ALE meta-analysis

Cluster[1]	Region[2]	Hem	BA[3]	x	y	z[4]	Volume[5]	PeakALEValue[6]
1	ParahippocampalGyrus	R	35	24	-38	-8	12208	0.08348948
	PosteriorCingulate	R	30	16	-54	16		0.04141214
	CerebellumAnterior	R		10	-48	4		0.037785392
	ParahippocampalGyrus	R	28	28	-24	-16		0.022493366
	CerebellumPosterior	R		30	-60	-16		0.02168812
2	PosteriorCingulate	L	30	-14	-58	12	8232	0.07428561
	CerebellumAnteriorVermis	R		4	-68	4		0.025932899
	LingualGyrus	L	18	-8	-72	-2		0.02222866
3	CerebellumAnterior	L		-24	-44	-14	7064	0.07554237
	ParahippocampalGyrus	L	35	-28	-30	-24		0.0320093
	ParahippocampalGyrus	L	27	-20	-36	-2		0.027473245
4	MiddleOccipitalGyrus	R	19	34	-76	18	6600	0.040562145
	SuperiorOccipitalGyrus	R	19	40	-76	32		0.036990047
	Precuneus	R	19	28	-72	44		0.022750996
5	Precuneus	L	7	-2	-64	56	5720	0.042710572
	Precuneus	R	7	8	-66	48		0.024276787
	SuperiorParietalLobule	L	7	-16	-62	60		0.023785586
	Precuneus	R	7	14	-64	56		0.023679676
6	FrontalSub-Gyral	R	6	26	6	54	5496	0.044161897
	MedialFrontalGyrus	L	6	-4	10	54		0.037767626
7	MiddleFrontalGyrus	L	6	-26	-2	54	3672	0.061935183
8	MiddleOccipitalGyrus	L	19	-32	-84	26	3056	0.0413119
9	Sub-lobarClaustrum	R		32	24	-4	1728	0.049173187
10	MiddleFrontalGyrus	R	10	28	56	-4	1616	0.0306352
11	CerebellumPosteriorPyramis	L		-8	-74	-26	1592	0.027504068
	CerebellumAnteriorPyramis	R		8	-70	-26		0.024822608
12	SuperiorParietalLobule	R	7	32	-56	52	1560	0.023125304
13	Sub-lobarInsula	L	13	-32	24	-2	1232	0.039251123
14	SuperiorParietalLobule	L	7	-30	-62	54	1120	0.021252666
	InferiorParietalLobule	L	40	-34	-54	46		0.021210987
	ParietalSub-Gyral	L	40	-34	-40	42		0.019823251
15	InferiorFrontalGyrus	R	9	50	12	30	1080	0.03151398
16	PrecentralGyrus	L	6	-48	6	34	792	0.02866637
17	Cuneus	L	7	-16	-74	38	648	0.022838764
	Precuneus	L	7	-22	-66	36		0.016088631
18	MiddleFrontalGyrus	R	9	46	28	24	568	0.02148259
19	SublobarThalamusMedialDorsalNucleu	R		8	-18	10	440	0.022488715
20	SublobarCaudateBody	R		14	4	14	296	0.022470044
21	MiddleOccipitalGyrus	L	19	-44	-80	12	280	0.021322485
22	BrainstemMidbrainRedNucleus	R		4	-24	-4	272	0.020608215
23	SupramarginalGyrus	R	40	54	-50	36	248	0.021613117
24	SublobarThalamusMedialDorsalNucleus	L		-10	-16	10	216	0.019071871
25	Precuneus	R	7	26	-64	36	216	0.018560035

[1]Number of clusters
[2]Region
[3]Brodmann's areas (if applicable)
[4]MNI coordinates of each foci
[5]Volume of cluster (mm^3)
[6]ALE value of each peak.
