# Supplementary material for: Neuropsychology of Environmental Navigation in Humans: Review and Meta-Analysis of fMRI Studies in Healthy Participants
Source: Neuropsychol Rev. 2014 Feb 1;24(2):236–51. doi: 10.1007/s11065-014-9247-8 (PMC4010721; doi:10.1007/s11065-014-9247-8)
Supplement: Supplementary file 2 — Results of ALE meta-analysis on familiar environments (PDF 22 kb) [file 11065_2014_9247_MOESM2_ESM.pdf]

**Table S2.** Results of ALE meta-analysis on familiar environments

| Cluster[1] | Region[2] | Hem | BA[3] | x | y | z[4] | Volume[5] | PeakALEValue[6] |
|---|---|---|---|---|---|---|---|---|
| 1 | PosteriorCingulate | L | 30 | -14 | -60 | 14 | 4976 | 0.045202162 |
| 2 | MiddleOccipitalGyrus | R | 19 | 34 | -76 | 18 | 4456 | 0.028292235 |
| | SuperiorOccipitalGyrus | R | 19 | 40 | -78 | 28 | | 0.026615687 |
| 3 | ParahippocampalGyrus | R | 35 | 24 | -36 | -10 | 3328 | 0.028204102 |
| | ParahippocampalGyrus | R | 30 | 12 | -44 | 4 | | 0.01488898 |
| | ParahippocampalGyrus | R | 30 | 14 | -50 | 6 | | 0.013950102 |
| | PosteriorCingulate | R | 29 | 16 | -50 | 16 | | 0.013536854 |
| 4 | MiddleFrontalGyrus | L | 6 | -26 | -4 | 54 | 3120 | 0.037932474 |
| | LobeSub-Gyral | L | 6 | -22 | 12 | 54 | | 0.013079222 |
| 5 | SuperiorOccipitalGyrus | L | 19 | -32 | -84 | 30 | 2424 | 0.028755322 |
| 6 | ParahippocampalGyrus | L | 36 | -28 | -44 | -12 | 1944 | 0.02899567 |
| 7 | ParahippocampalGyrus | L | 35 | -28 | -28 | -22 | 984 | 0.021456186 |
| 8 | SuperiorTemporalGyrus | L | 22 | -54 | -18 | -2 | 944 | 0.016797045 |
| 9 | SuperiorFrontalGyrus | L | 6 | 0 | 18 | 58 | 416 | 0.013609743 |
| 10 | CerebellumPosteriorLobeCerebellar | L | | 0 | -54 | -38 | 352 | 0.016327094 |
| 11 | SuperiorTemporalGyrus | R | 22 | 54 | -36 | 0 | 344 | 0.012032246 |
| 12 | CingulateGyrus | R | 24 | 18 | 14 | 48 | 336 | 0.013055553 |
| 13 | Sub-lobarInsula | L | 13 | -38 | 24 | -4 | 320 | 0.014524751 |
| 14 | Precuneus | R | 7 | 28 | -70 | 44 | 312 | 0.014506971 |
| 15 | CerebellumPosteriorLobeDeclive | L | | -18 | -84 | -12 | 304 | 0.014783121 |
| 16 | InferiorFrontalGyrus | L | 44 | -58 | 8 | 12 | 304 | 0.014965232 |
| 17 | ParahippocampalGyrus | R | 35 | 26 | -26 | -20 | 288 | 0.013433669 |
| 18 | Precuneus | R | 7 | 8 | -54 | 58 | 272 | 0.014254489 |
| 19 | SuperiorTemporalGyrus | R | 39 | 52 | -52 | 6 | 264 | 0.014401713 |
| 20 | SublobarCaudateBody | L | | -2 | 16 | 6 | 256 | 0.01366244 |
| 21 | MiddleFrontalGyrus | R | 9 | 48 | 28 | 26 | 248 | 0.014239919 |
| 22 | InferiorParietalLobule | L | 40 | -46 | -46 | 50 | 240 | 0.01106057 |
| 23 | AnteriorCingulate | R | 32 | 10 | 38 | 14 | 216 | 0.013148258 |

[1]Number of clusters
[2]Region
[3]Brodmann's areas (if applicable),
[4]MNI coordinates of each foci,
[5]Volume of cluster ($mm^3$)
[6]ALE value of each peak.
