# Supplementary material for: Neuropsychology of Environmental Navigation in Humans: Review and Meta-Analysis of fMRI Studies in Healthy Participants
Source: Neuropsychol Rev. 2014 Feb 1;24(2):236–51. doi: 10.1007/s11065-014-9247-8 (PMC4010721; doi:10.1007/s11065-014-9247-8)
Supplement: Supplementary file 3 — Results of ALE meta-analysis on recently learned environments (PDF 25 kb) [file 11065_2014_9247_MOESM3_ESM.pdf]

Table S3. Results of ALE meta-analysis on recently learned environments

Cluster[1]	Region[2]	Hem	BA[3]	x	y	z[4]	Volume[5]	PeakALEValue[6]
1	ParahippocampalGyrus	R	35	24	-38	-8	9936	0.06153264
	PosteriorCingulate	R	30	18	-54	16		0.032647964
	CerebellumAnteriorLobeCulmen	R		8	-48	2		0.027573392
	BrainstemMidbrainRedNucleus	R		4	-24	-4		0.020608177
2	Precuneus	L	7	-2	-66	56	5928	0.03686537
	Precuneus	R	7	8	-66	48		0.024037667
	Precuneus	R	7	14	-64	54		0.023583543
	SuperiorParietalLobule	L	7	-16	-60	60		0.021888962
3	CerebellumAnteriorLobeCulmen	L		-22	-44	-14	5552	0.05969538
	ParahippocampalGyrus	L	27	-20	-34	-2		0.026041834
	Hippocampus	L		-32	-40	-4		0.021709582
4	PosteriorCingulate	L	30	-14	-56	16	3464	0.034938175
5	MedialFrontalGyrus	L	6	-4	8	54	2552	0.035363827
6	MiddleOccipitalGyrus	L	19	-32	-84	24	2344	0.024355497
	Cuneus	L	7	-16	-74	38		0.02282314
	SuperiorOccipitalGyrus	L	19	-28	-78	34		0.020483425
	Precuneus	L	7	-22	-66	36		0.016086198
7	Precuneus	R	19	38	-74	38	2176	0.023646748
	MiddleOccipitalGyrus	R	19	40	-80	18		0.017866569
	MiddleOccipitalGyrus	R	19	34	-80	28		0.016559603
8	LingualGyrus	R	18	4	-70	4	2024	0.024900563
	LingualGyrus	R	18	-8	-72	-2		0.02220561
9	SublobarClaustrum	R		32	24	-4	1848	0.049052637
10	FrontalLobeSub-Gyral	L	6	-28	0	58	1792	0.032189462
11	FrontalLobeSub-Gyral	R	6	26	6	54	1760	0.038251974
12	SuperiorParietalLobule	R	7	32	-56	52	1736	0.022994647
13	CerebellumPosteriorLobe	L		-6	-74	-28	1728	0.023406716
	CerebellumAnteriorLobePyramis	R		8	-70	-26		0.021047527
14	MiddleFrontalGyrus	R	10	28	56	-4	1712	0.030357558
15	SublobarClaustrum	L		-30	24	-4	1016	0.034571767
16	InferiorFrontalGyrus	R	9	48	12	30	1000	0.024678761
17	ParietalLobe	L	40	-34	-40	42	752	0.019796781
	InferiorParietalLobule	L	40	-34	-52	46		0.017041767
18	SublobarThalamusMedialDorsalNucleus	R		8	-18	10	704	0.022487706
19	SuperiorParietalLobule	L	7	-30	-62	54	664	0.02092932
20	SublobarCaudateBody	R		14	4	14	456	0.022469979
21	Precuneus	R	7	26	-64	36	376	0.01820192
22	MiddleOccipitalGyrus	L	19	-44	-80	12	376	0.020744363
23	InferiorFrontalGyrus	L	6	-48	6	32	328	0.01908378
24	ParahippocampalGyrus	L	35	-20	-22	-14	320	0.016290804
25	SublobarCaudateBody	L		-8	6	14	320	0.020168103
26	SuperiorFrontalGyrus	R	9	44	44	22	272	0.015909562

[1]Number of clusters
[2]Region
[3]Brodmann's areas (if applicable),
[4]MNI coordinates of each foci,
[5]Volume of cluster (mm^3)
[6]ALE value of each peak.
