# Supplementary material for: Neuropsychology of Environmental Navigation in Humans: Review and Meta-Analysis of fMRI Studies in Healthy Participants
Source: Neuropsychol Rev. 2014 Feb 1;24(2):236–51. doi: 10.1007/s11065-014-9247-8 (PMC4010721; doi:10.1007/s11065-014-9247-8)
Supplement: Supplementary file 4 — Results of ALE meta-analysis on Allocentric studies (PDF 21 kb) [file 11065_2014_9247_MOESM4_ESM.pdf]

**Table S4.** Results of ALE meta-analysis on Allocentric studies

| Cluster[1] | Region[2] | Hem | BA[3] | x | y | z[4] | Volume[5] | PeakALEValue[6] |
|---|---|---|---|---|---|---|---|---|
| 1 | ParahippocampalGyrus | R | 27 | 24 | -36 | -6 | 3872 | 0.034848787 |
| | ParahippocampalGyrus | R | 30 | 16 | -52 | 6 | | 0.017341634 |
| | PosteriorCingulate | R | 30 | 20 | -58 | 18 | | 0.017219866 |
| | CerebellumAnteriorCulmen | R | | 8 | -50 | 0 | | 0.012071343 |
| 2 | PosteriorCingulate | L | 30 | -14 | -58 | 12 | 3608 | 0.041504808 |
| 3 | ParahippocampalGyrus | L | 36 | -24 | -46 | -12 | 2208 | 0.03315161 |
| 4 | Precuneus | L | 7 | -2 | -66 | 54 | 2184 | 0.030753713 |
| | Precuneus | L | 7 | -14 | -64 | 54 | | 0.018959673 |
| 5 | MiddleOccipitalGyrus | R | 19 | 40 | -78 | 20 | 1760 | 0.019456368 |
| | MiddleTemporalGyrus | R | 39 | 46 | -70 | 26 | | 0.017588288 |
| 6 | MiddleOccipitalGyrus | L | 19 | -32 | -84 | 24 | 1656 | 0.023848165 |
| 7 | MiddleFrontalGyrus | L | 6 | -26 | -4 | 54 | 1320 | 0.028820183 |
| 8 | ParahippocampalGyrus | L | 35 | -28 | -30 | -24 | 1168 | 0.029307768 |
| 9 | InferiorFrontalGyrus | R | 9 | 50 | 12 | 30 | 1008 | 0.023238456 |
| 10 | FrontalSub-Gyral | R | 6 | 26 | 4 | 56 | 640 | 0.01962117 |
| 11 | SuperiorTemporalGyrus | L | 22 | -54 | -18 | -2 | 568 | 0.016797049 |
| 12 | Precuneus | R | 7 | 28 | -72 | 46 | 472 | 0.018729068 |
| 13 | MiddleFrontalGyrus | R | 9 | 46 | 28 | 24 | 376 | 0.015462393 |
| 14 | PrecentralGyrus | L | 6 | -48 | 6 | 36 | 368 | 0.017950112 |
| 15 | MiddleFrontalGyrus | R | 10 | 28 | 56 | -4 | 352 | 0.015372936 |
| 16 | MiddleFrontalGyrus | R | 9 | 28 | 38 | 30 | 264 | 0.016630342 |
| 17 | SuperiorParietalLobule | R | 7 | 34 | -48 | 46 | 264 | 0.013873719 |
| 18 | LingualGyrus | R | 18 | 20 | -76 | -2 | 240 | 0.01536322 |
| 19 | CerebellumPosteriorCerebellar | L | | 0 | -54 | -38 | 224 | 0.016330406 |
| 20 | CerebellumPosteriorDeclive | R | | 30 | -60 | -16 | 224 | 0.014764383 |
| 21 | SublobarClaustrum | R | | 32 | 24 | -4 | 208 | 0.015184716 |
| 22 | SublobarInsula | L | | -32 | 24 | -4 | 208 | 0.014145426 |

[1]Number of clusters
[2]Region
[3]Brodmann's areas (if applicable),
[4]MNI coordinates of each foci,
[5]Volume of cluster (mm$^3$)
[6]ALE value of each peak.
