# Supplementary material for: Neuropsychology of Environmental Navigation in Humans: Review and Meta-Analysis of fMRI Studies in Healthy Participants
Source: Neuropsychol Rev. 2014 Feb 1;24(2):236–51. doi: 10.1007/s11065-014-9247-8 (PMC4010721; doi:10.1007/s11065-014-9247-8)
Supplement: Supplementary file 5 — Results of ALE meta-analysis on Egocentric studies (PDF 23 kb) [file 11065_2014_9247_MOESM5_ESM.pdf]

Table S5. Results of ALE meta-analysis on Egocentric studies

Cluster[1]	Region[2]	Hem	BA[3]	x	y	z[4]	Volume[5]	PeakALEValue[6]
1	ParahippocampalGyrus	R	35	24	-38	-10	8376	0.05278939
	CerebellumAnteriorCulmen	R		10	-46	4		0.03126464
	PosteriorCingulate	R	30	16	-52	16		0.030800309
2	SuperiorOccipitalGyrus	R	19	40	-74	34	5048	0.033761524
	MiddleOccipitalGyrus	R	19	34	-76	18		0.02311313
3	ParahippocampalGyrus	L	36	-26	-44	-12	4888	0.03505675
	ParahippocampalGyrus	L	27	-20	-34	0		0.018663136
4	PosteriorCingulate	L	30	-14	-58	16	4664	0.03788363
	PosteriorCingulate	L	30	-12	-56	10		0.03657413
5	MiddleFrontalGyrus	L	6	-28	-2	56	3096	0.038042728
6	Precuneus	L	7	2	-62	58	2496	0.021642948
	Precuneus	R	7	8	-66	48		0.01981026
7	SuperiorOccipitalGyrus	L	19	-30	-80	32	2472	0.021241834
	Cuneus	L	7	-16	-76	38		0.01646159
	Precuneus	L	7	-22	-66	36		0.015603563
8	MedialFrontalGyrus	L	6	-4	10	54	2456	0.031735092
9	CerebellumAnteriorCulmenofVermis	R		4	-68	4	1472	0.021861713
	LingualGyrus	L	18	-8	-74	0		0.017421197
10	SublobarInsula	R	13	34	26	-2	1184	0.031131027
11	FrontalSub-Gyral	R	6	26	6	52	992	0.020920258
	MiddleFrontalGyrus	R	6	30	-2	54		0.015841292
12	CerebellumPosteriorPyramis	L		-8	-74	-26	936	0.026147805
13	CerebellumAnteriorPyramis	R		8	-70	-26	664	0.020944906
14	SuperiorParietalLobule	L	7	-30	-62	52	600	0.019086966
15	SuperiorFrontalGyrus	R	10	28	56	4	552	0.01940248
16	SublobarInsula	L	13	-34	24	-2	544	0.020549603
17	SublobarCaudateCaudateBody	R		14	4	12	448	0.021093262
18	SuperiorParietalLobule	L	7	-16	-60	62	400	0.01724958
19	ParahippocampalGyrus	R	34	18	-14	-20	304	0.013858027
	ParahippocampalGyrusAmygdala	R		20	-10	-16		0.013280268
20	InferiorOccipitalGyrus	L	18	-30	-90	-6	296	0.01510856
21	Sub-lobarThalamus	L		-12	-16	10	288	0.013717365
22	MiddleFrontalGyrus	L	46	-46	26	24	272	0.015835201
23	MiddleTemporalGyrus	L	39	-46	-74	32	272	0.015996706
24	SuperiorFrontalGyrus	R	9	44	44	22	248	0.014549848
25	MiddleFrontalGyrus	L	10	-34	48	6	208	0.016235163

[1]Number of clusters
[2]Region
[3]Brodmann's areas (if applicable),
[4]MNI coordinates of each foci,
[5]Volume of cluster (mm^3)
[6]ALE value of each peak.
