# Supplementary material for: Neuropsychology of Environmental Navigation in Humans: Review and Meta-Analysis of fMRI Studies in Healthy Participants
Source: Neuropsychol Rev. 2014 Feb 1;24(2):236–51. doi: 10.1007/s11065-014-9247-8 (PMC4010721; doi:10.1007/s11065-014-9247-8)
Supplement: Supplementary file 6 — Contrast analysis between different strategies (Allocentric vs. Egocentric) and different paradigms (Recently learned vs. Familiar) (PDF 54.7 kb) [file 11065_2014_9247_MOESM6_ESM.pdf]

Table S6. Contrast analysis between different strategies (Allocentric *vs.* Egocentric) and different paradigms (Recently learned *vs.* Familiar)

Cluster[1]	Region	Hem	BA[2]	Volume[3]	Weighted Center (x,y,z)[4]			Extrema Value[5]
Strategies								
Allo *vs.* Ego								
No suprathreshold cluster								
Ego *vs.* Allo								
1	Superior Occipital Gyrus	R	39	1040	39.03	73.65	37.29	3.431614
	Angular Gyrus	R	39					3.194651
	Precuneus	R	19					3.155907
Paradigms								
F *vs.* RL								
1	Posterior Cingulate	L	30	768	-8.24	-60.41	11.35	3.8905919
2	Middle Temporal Gyrus	R	19	440	39.71	-73.77	23.42	2.758879
3	Middle Frontal Gyrus	L	6	416	-22.6	-3.2	50.72	3.1213892
	Middle Frontal Gyrus	L	6					2.967738
4	Middle Temporal Gyrus	R		344	55.01	-35.27	-0.99	3.7190166
RL *vs.* F								
1	Precuneus	R	7	1264	11.67	-65.67	52.7	3.719017
	Precuneus	R	7					3.431614
	Precuneus	R	7					3.290527
2	Sublobar.Claustrum	R		776	30.13	25.25	-2.15	3.090232
	Insula	R	13					3.061814
	Sublobar.Claustrum	R						2.988882
3	Inferior Parietal Lobule	R	40	728	33.36	-54.01	46.35	3.890592
	Precuneus	R	7					3.23888
4	Cuneus	L	7	696	-18.02	-70.5	37.52	3.890592
	Precuneus	L	7					3.431614
5	Brainstem Midbrain	L		688	7.83	-24.97	-1.66	3.890592
6	Sublobar Thalamus	R		520	8.23	-16.84	8.36	3.890592
	Sublobar Thalamus	R						3.719017
	Sublobar Thalamus	R						3.540084
7	Anterior Cerebellum	L		304	-9.24	-73.22	-1.8	2.911238
	Lingual Gyrus	L	18					2.833787
8	Parahippocampal Gyrus	R	19	272	30.44	-45.32	-7.12	3.194651

[1]Number of clusters
[2]Region, Brodmann's areas (if applicable)
[3]Volume of cluster (mm^3)
[4]Weighted center of MNI coordinates of each foci
[5]Extrema value.
